# How the Plant Temperature Links to the Air Temperature in the Desert Plant *Artemisia ordosica*

**DOI:** 10.1371/journal.pone.0135452

**Published:** 2015-08-17

**Authors:** Ming-Han Yu, Guo-Dong Ding, Guang-Lei Gao, Bao-Ping Sun, Yuan-Yuan Zhao, Li Wan, De-Ying Wang, Zi-Yang Gui

**Affiliations:** 1 Yanchi Research Station, School of Soil and Water Conservation, Beijing Forestry University, Beijing, 100083, China; 2 Key Laboratory of Soil and Water Conservation and Desertification Combating, Ministry of Education, Beijing Forestry University, Beijing, 100083, China; Universidade Federal de Viçosa, BRAZIL

## Abstract

Plant temperature (Tp) is an important indicator of plant health. To determine the dynamics of plant temperature and self-cooling ability of the plant, we measured Tp in *Artemisia ordosica* in July, in the Mu Us Desert of Northwest China. Related factors were also monitored to investigate their effects on Tp, including environmental factors, such as air temperature (Ta), relative humidity, wind speed; and physiological factors, such as leaf water potential, sap flow, and water content. The results indicate that: 1) Tp generally changes in conjunction with Ta mainly, and varies with height and among the plant organs. Tp in the young branches is most constant, while it is the most sensitive in the leaves. 2) Correlations between Tp and environmental factors show that Tp is affected mainly by Ta. 3) The self-cooling ability of the plant was effective by midday, with Tp being lower than Ta. 4) Increasing sap flow and leaf water potential showed that transpiration formed part of the mechanism that supported self-cooling. Increased in water conductance and specific heat at midday may be additional factors that contribute to plant cooling ability. Therefore, our results confirmed plant self-cooling ability. The response to high temperatures is regulated by both transpiration speed and an increase in stem water conductance. This study provides quantitative data for plant management in terms of temperature control. Moreover, our findings will assist species selection with taking plant temperature as an index.

## Introduction

Plant temperature (Tp) is an important physiological and ecological characteristic of plants and is a result of the interaction between the external environment and internal adjustment mechanisms (the energy exchange between the plant and its environment) [1]. Tp affects myriad complex life activities, including enzyme reactions, membrane transport, transpiration, and is a key indicator of plant health [2]. Thus, it is important to study the mechanisms of regulation of Tp, which have become a topic of considerable interest in recent studies [3, 4].

Many studies of plant growth have used the measured air temperature (Ta) as an index to determine Tp [5]. However, numerous studies indicate differences in the response of Tp when Ta changes by only a few degrees Celsius [6]. When plants grow under conditions of strong solar and thermal radiation exchange, they are often warmer than the air during the day and cooler than the air at night [7]. The adaptations for species’ survival in environments of intense heat and water stress are closely related to its accommodative responses. The adaptation of self-cooling is one of the most significant of these responses. Thus, the measurement of Tp can aid the selection of drought and heat-resistant species for vegetation in areas of desertification. The monitoring of Tp represents a quantitative method to better understand how plants respond to their environment and to guide decision-making regarding vegetation management. Although previous studies have shown that plants have evolved mechanisms to achieve some degree of self-cooling ability, plant self-cooling is still poorly understood [8, 9].

The leaf is the most common target for Tp studies; because it is the most sensitive organ and can easily change their morphology and inner- structure to adapt to the existing conditions [10]. Many studies have focused on variation in leaf temperature, especially with transpiration. Calculations of energy balance show that if the transpiration of plant leaves decreases, the decrease in latent heat exchange will result in an increase in Tp [11]. For example, estimates from data from Tanner for a representative day at midday in early September indicate that a decrease in transpiration of 10% from a full coverage of alfalfa would cause a temperature increase of 1.0°C approximately [12]. However, other organs such as the stems are also important for plant growth, and their temperature should also be considered. As the main organ for biomass heat storage in a forest canopy, the stem is also a key part of the plant that senses the ambient environment and energy exchange besides the leaf [13]. The xylem in the stem is responsible for water delivery, and stem temperature affects the transportation efficiency of the water. Studies of Tp in the stem provide important information for eluding the mechanisms affecting Tp. However, such studies are rare because of the difficulty of measuring stem temperature.

We chose *Artemisia ordosica* to investigate the regulation of stem temperature, especially in arid and semi-arid regions where water resources are lacking [14]. *Artemisia ordosica* is the most common species in arid and semi-arid North China. It has been widely used in vegetation restoration in areas suffering desertification and has survived successfully. Because of its widespread occurrence, hardiness and longevity, we chose this species as a model in desert environments. The purpose of this study was to measure the temperature in *Artemisia ordosica* in the leaves and stems, record related environmental factors, and analyze certain features of the mechanism causing variations in Tp. The objectives of the study were as follows:

1. To explore how different organs of the plant regulate Tp both spatially and temporally.
2. To determine the roles of different factors in regulating Tp.
3. To confirm the existence of plant self-cooling and how internal controls determine this ability.

## Materials and Methods

### Field experiment site

The study site is managed by the Beijing Forestry University and Environment Protection and Forestry Bureau of Yanchi County and does not contain any national park or other protected area of land or sea. The Environment Protection and Forestry Bureau of Yanchi County supervised the protection of wildlife and environment during the fieldwork. The location is not privately owned or protected. No specific permits were required for the field studies. Yanchi Research Station was founded by Beijing Forestry University and authorized by the Chinese government. The authorities and the authors confirm that the field studies did not involve endangered or protected species.

The fieldwork was carried out on the top of a sand dune at the Yanchi Research Station in the Autonomous Region of Ningxia (37°04´–38°10´N, 106°30´–107°41´E), part of the Mu Us Desert in China in July 2014. The station is located in a semi-arid region that is generally warm and dry, with daytime air temperatures and relative humidity (RH) ranging from -29.4–37.5°C and 49–55%, respectively. The mean annual precipitation is around 292 mm. Precipitation is concentrated from July to September (with more rain falling in August), which accounts for 60% to 75% of the annual precipitation. Mean annual potential transpiration is 2024 mm, about threefold the mean annual precipitation. The soil type is aeolian sandy. The plot was chosen on flat ground and vegetation within the plot had been irrigated by natural rainfall since its first planting in 2011. Plant coverage was 70%, and the area of the plot was 400 m^2^.

### Observations of Tp and microclimate

All Tp and microclimate data were collected automatically every 10 s and stored every 10 min. The data were collected and processed in real time to provide near-continuous measurements.

Tp was measured using thermocouple thermometers (L-95, Ya Xin, China) with sensors inserted into the xylems or leaves at fractions from the bottom of 0,1/4,1/2,3/4, and 1 of the total branch height. Two biennial plants were included. The copper constantan thermocouples were held firmly in place by taping them around the stems.

Ta and RH were measured with a thermohygrometer (HMP155A, Vaisala, Finland) on a flux tower at 6 m above ground.

Wind velocity (V) was determined using a Solent 3D ultrasonic anemometer (R2 Gill Instruments, Lymington, UK) at a height of 6 m above ground on the flux tower.

Solar radiation (Rs) was measured using a four-component radiometer (CNR-4, Kipp & Zonen, The Netherlands) on the flux tower, also at 6 m above ground.

The plant crown and soil temperatures (Tc and Ts) were measured using thermocouple thermometers (L-95 Ya Xin, China) with sensors fixed at heights of 10 cm and 5 cm below the soil surface and the sensors were fixed at fractions from the main stem of 0, 1/4, 1/2, 3/4, and 1 of the total branch lengths to measure the temperatures of the air and soil.

Relative humidity in the crown (Hc) was measured using a thermohygrometer (L-95, Ya Xin, China) both inside and outside of the crown at half or the total height of the plant.

Vapor pressure deficit (VPD), which is associated with stomatal and hydraulic conductance, was calculated from Eq (1). As demonstrated by previous studies, transpiration velocity increases when VPD increases, which means that more energy will be removed through transpiration [15–17].

$$VPD=6.107\times e^{\frac{17.4Ta}{239+Ta}}\times \left(100-RH\right).$$ (1)

### Measurement of physiological characteristics

To ascertain the mechanisms determining how plants maintain a limited temperature range in terms of evaporation and heat capacity, we measured various water and heat related physiological characteristics, as follows:

The leaf water potential was measured using a water potential apparatus (HAD-PSYPRO, WESCOR, American) once every hour on clear days, so that 24 data points were acquired per day in each of three leaves from different branches.

The sap flow rate was measured continuously using a sap flow system (EMS 62, Ecliptek, Switzerland).

The water content was measured every hour in three leaf samples and five stems by weighing.

The water conductance of the stem was calculated by dividing the sap flow by the difference in leaf water potential measured before dawn and at the given time [18].

### Data filtering and processing

To exclude the precipitation from influencing the accuracy of the results, we selected data from clear days for analysis. All of the selected days had wind speeds that varied from 2–3 m/sec, and were clear and sunny. The air temperature range was 10°C– 35°C, and the humidity was between 50% and 55%. No condensation occurred on the leaves at night.

Statistical data analysis was carried out using the SPSS statistics version 18 software. Data were tested for normality and homogeneity of variations using Kolmogorav–Smirnov and Levene’s tests, respectively. An analysis of the variation between Tp and Ta was performed using one-way repeated measures. The effects of microclimate factors on Tp were analyzed using an analysis of covariance (ANOVA). The significance of the relationships between Tp and the environmental factors was assessed using Pearson’s correlation. Regression analysis was used to identify significant relationships between Tp and sap flow, leaf water potential, and water conductance.

Plant temperature, physiological characteristics and environmental variable data from the present study are presented in S1 Dataset.

## Results

### Dynamics of Tp variation

#### Temporal dynamics of Tp

**Fig 1:**
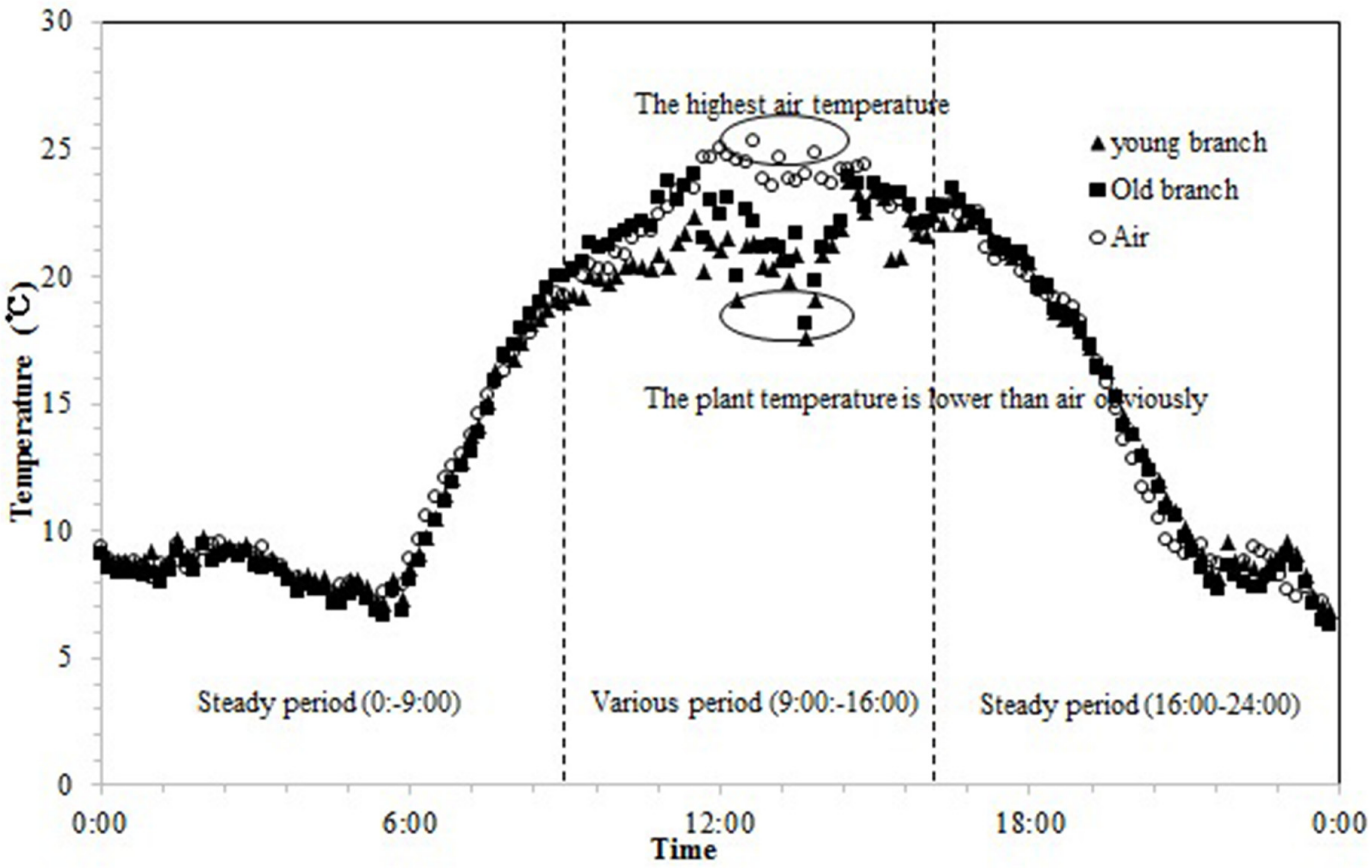
Daily variation in plant temperature (Tp) of *Artemisia ordosica* on clear days from 5 July to 11 July 2014. A marked difference between Ta and Tp was observed during the period between 9:00 and 16:00.

As shown in Fig 1, Tp generally varies in conjunction with Ta. Tp was fairly low from 18:00 to 6:00 (during the night), and increased to 25°C at midday. When the Tp in both old and young branches was analyzed relative to Ta, the results from Mauchly’s test (Table 1) indicated that the assumption of sphericity had been violated, χ^2^(3) = 271.371 where p = 0.00; therefore, the number of degrees of freedom were corrected using Greenhouse-Geisser estimates of sphericity. Tests of within-subject effects (F (1.53, 1152.20) = 57.03, p = 0.00) suggested that Tp was significantly different from Ta (Table 2). The most significant variation appeared at midday, as shown in Fig 1. The maximum value of Tp occurred at 12:00, 1 or 2 h earlier than that of Ta. The minimum values of both Tp and Ta, however, occurred around 6:00.

**Table 1:** Mauchly’s test of sphericity for testing raw data. Mauchly's test of sphericity was examined to test for equality of variance. Since the Sig. probability (.000) is less than .05, the variances among the three sets of scores are not equal, which means that a statistical correction is necessary due to the lack of homogeneity (equality) of variance.

Measure:MEASURE_1							
Within Subjects Effect	Mauchly’s W	Approx.Chi-Squared	df	Sig.	Greenhouse-Geisser	Huynh-Feldt	Lower bound
Temperature	0.696	271.371	2	0.000	0.767	0.768	0.500

**Table 2:** Tests of within-subjects effects between air temperature (Ta) and plant temperature (Tp) in *Artemisia ordosica*. The test result (F (1.53, 1152.20) = 57.03, p = 0.00) suggested that Tp was significantly different from Ta.

Measure: MEASURE_1						
Source		Type III Sum of Squares	df	Mean Square	F	Sig.
Page	Sphericity Assumed	267.872	2	133.936	57.032	.000
	Greenhouse-Geisser	267.872	1.534	174.599	57.032	.000
	Huynh-Feldt	267.872	1.537	174.312	57.032	.000
	Lower-bound	267.872	1.000	267.872	57.032	.000
Error (page)	Sphericity Assumed	3527.340	1502	2.348		
	Greenhouse-Geisser	3527.340	1152.195	3.061		
	Huynh-Feldt	3527.340	1154.087	3.056		
	Lower-bound	3527.340	751.000	4.697		

#### Differences in Tp with height

**Fig 2:**
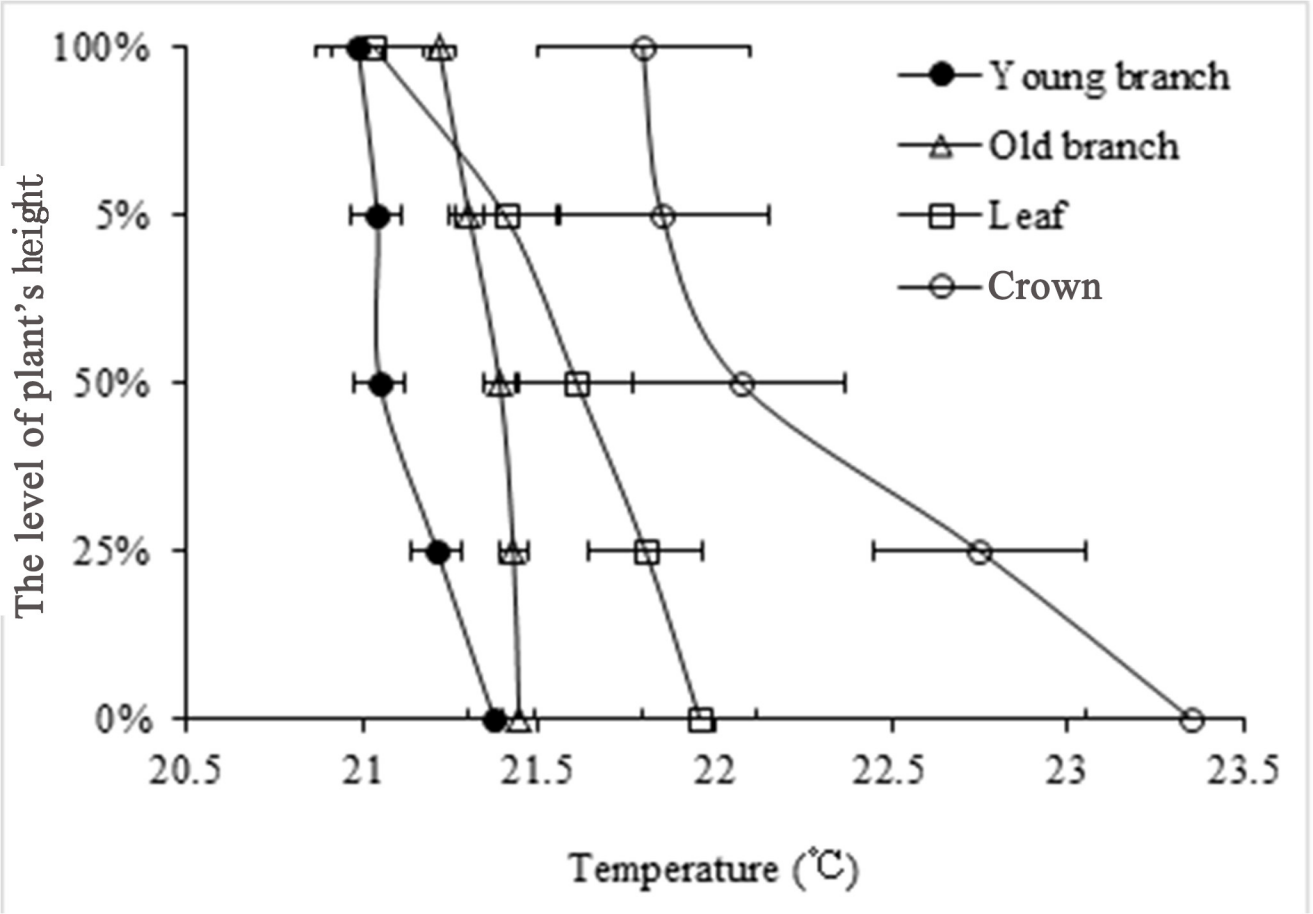
Differences in plant temperature (Tp) of various tissues of *Artemisia ordosica* at different heights in various plant organs.

Measurements of Tp at midday (9:00–16:00 when Tp is the most sensitive) showed that the Tp at the bottom of the plant was higher than that at the top, and similarly for Tc (Fig 2). The Tp at the fractions from the bottom of 0, 1/4, 1/2, 3/4, and 1 of the total height of the plant were lower than the Tc at the corresponding heights.

#### Differences in Tp among organs

**Fig 3:**
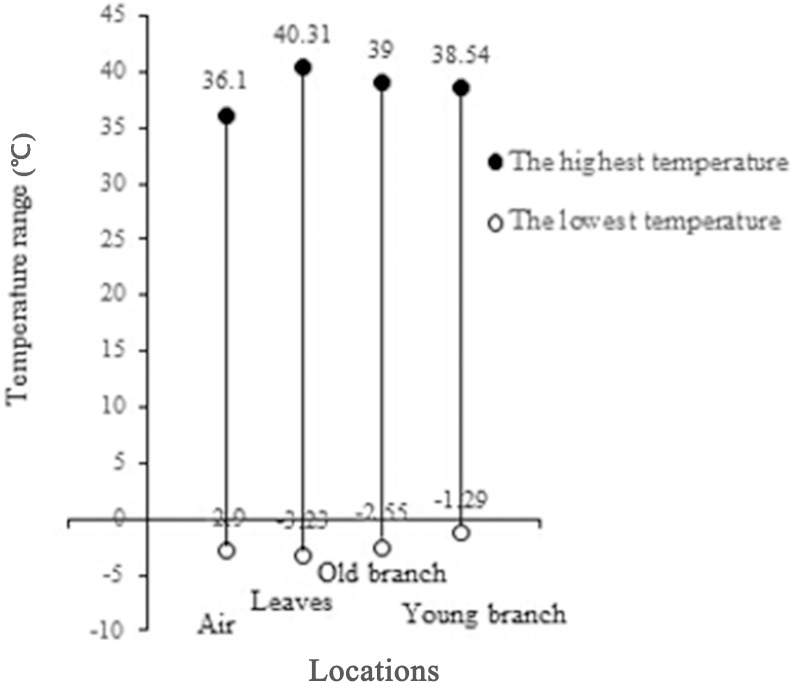
Temperature range of leaves, old branches and young branches of *Artemisia ordosica* during the course of a typical day. Air temperature (Ta) is also presented for comparison. Young branches demonstrated the narrowest temperature range and leaves the widest, showing that the sensitivity to temperature differs among tissues.

**Fig 4:**
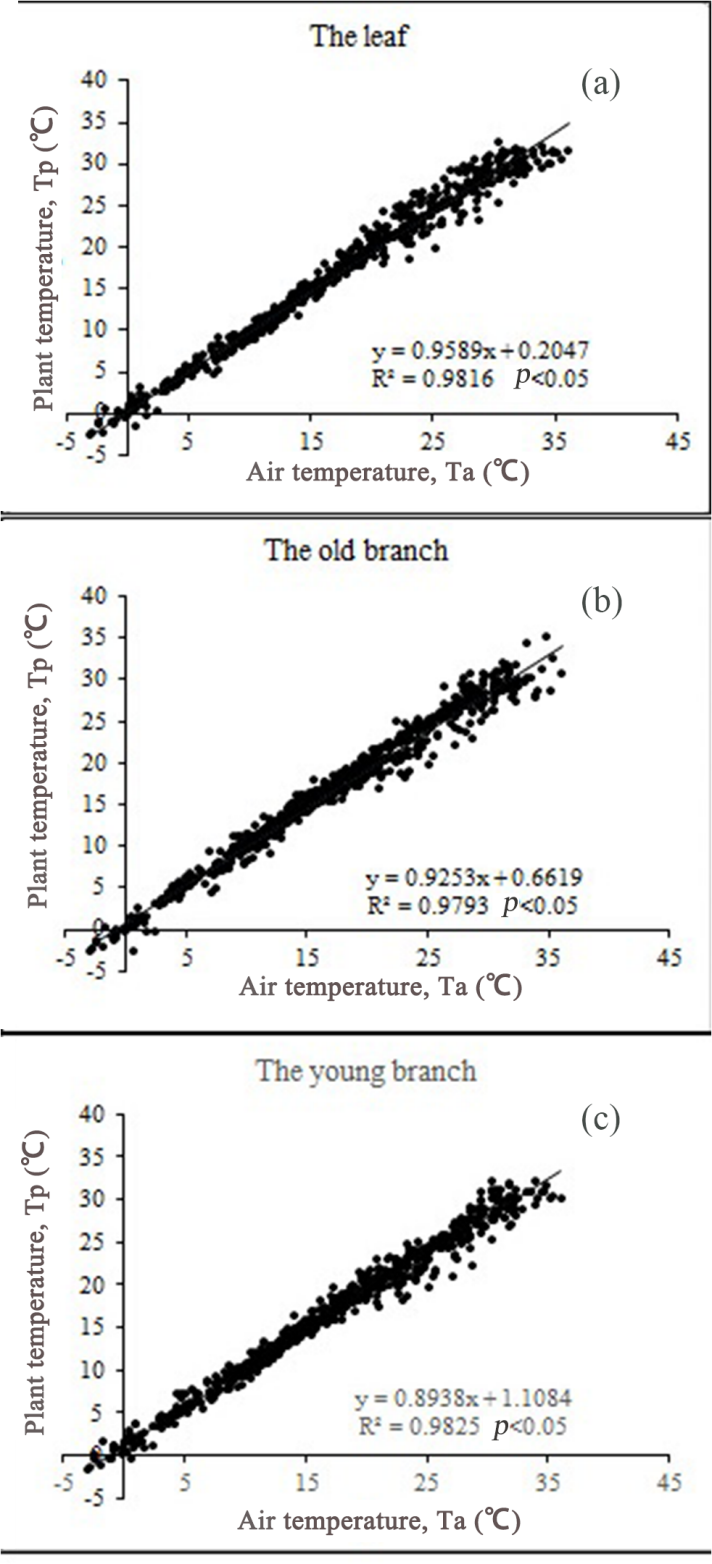
Correlations between plant temperature (Tp) and air temperature (Ta) in tissues of *Artemisia ordosica*. The slope of the line shows the sensitivity of Tp to Ta, leaves exhibited the greatest sensitivity and young branches the least.

The temperature variations in the leaves, old and young branches were clearly captured during the experiment (Fig 3). The temperature of the young branches fell within a narrow range, indicating that it was not markedly affected by the environment. Regression analysis between Tp and Ta (Fig 4), showed a higher slope value (0.9589) for the temperature of the leaves, indicating higher sensitivity in the leaves than in the old and young branches.

### Effects of environmental factors on Tp

The significant factors affecting Tp (Table 3) were Ta and Tc, with coefficients above 0.9. The other meteorological factors, RH, V, and S, typically had coefficients below 0.6, which is too small to influence Tp. Obviously, the Ta and Tc should be further investigated to better understand the variations in Tp.

**Table 3:** Correlations between plant temperature (Tp) in *Artemisia ordosica* and environmental factors. For all dates measurements were conductes over periods of up to 30 min and normalized before correlation analysis. Larger values indicate a stronger relationship with the corresponding index. Ta and Tc had larger values than Tp.

Parameter	Tp0	Tp1/4	Tp1/2	Tp3/4	Tp1	Ta	Tc	V	S	RH	Hc
Tp0	1.000	0.997	0.996	0.989	0.990	0.995	0.998	-0.348	0.489	-0.424	-0.597
Tp1/4		1.000	0.992	0.985	0.984	0.994	0.999	-0.325	0.504	-0.398	-0.577
Tp2/4			1.000	0.979	0.976	0.995	0.994	-0.401	0.587	-0.476	-0.549
Tp3/4				1.000	0.979	0.997	0.989	-0.422	0.566	-0.466	-0.520
Tp1					1.000	0.998	0.993	-0.459	0.619	-0.412	-0.498
Ta						1.000	0.956	-0.393	0.601	-0.496	-0.575
Tc							1.000	-0.565	0.475	-0.417	-0.592
V								1.000	-0.220	0.223	0.124
S									1.000	-0.300	-0.441
RH										1.000	0.899
Hc											1.000

N = 143. Tp0, Tp1/4, Tp1/2, Tp3/4, Tp1: Plant temperatures at fractions from the bottom of 0,1/4, 1/2, 3/4,1 of the total plant height; Ta: Air temperature; Tc: Air temperature in the crown; V: wind speed; S: solar radiation intensity; RH: Relative humidity; Hc: Relative humidity in the crown.

### Plant self-cooling ability

#### Phenomena of plant self-cooling

**Fig 5:**
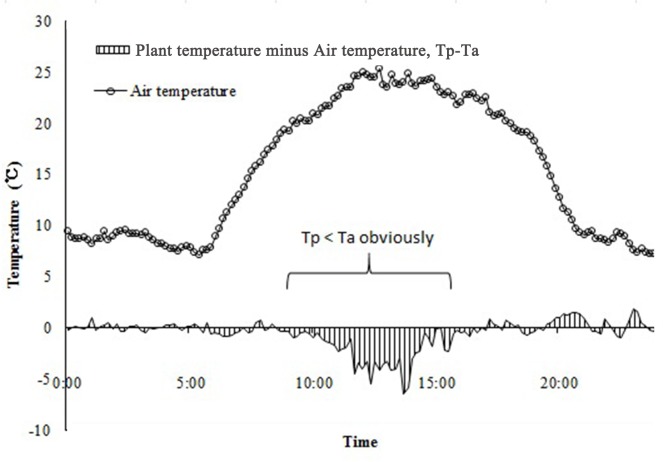
Characteristics of plant-air temperature differences at midday. Plant-air temperature differences in this figure were calculated as: plant temperature (Tp) minus air temperature (Ta).

**Fig 6:**
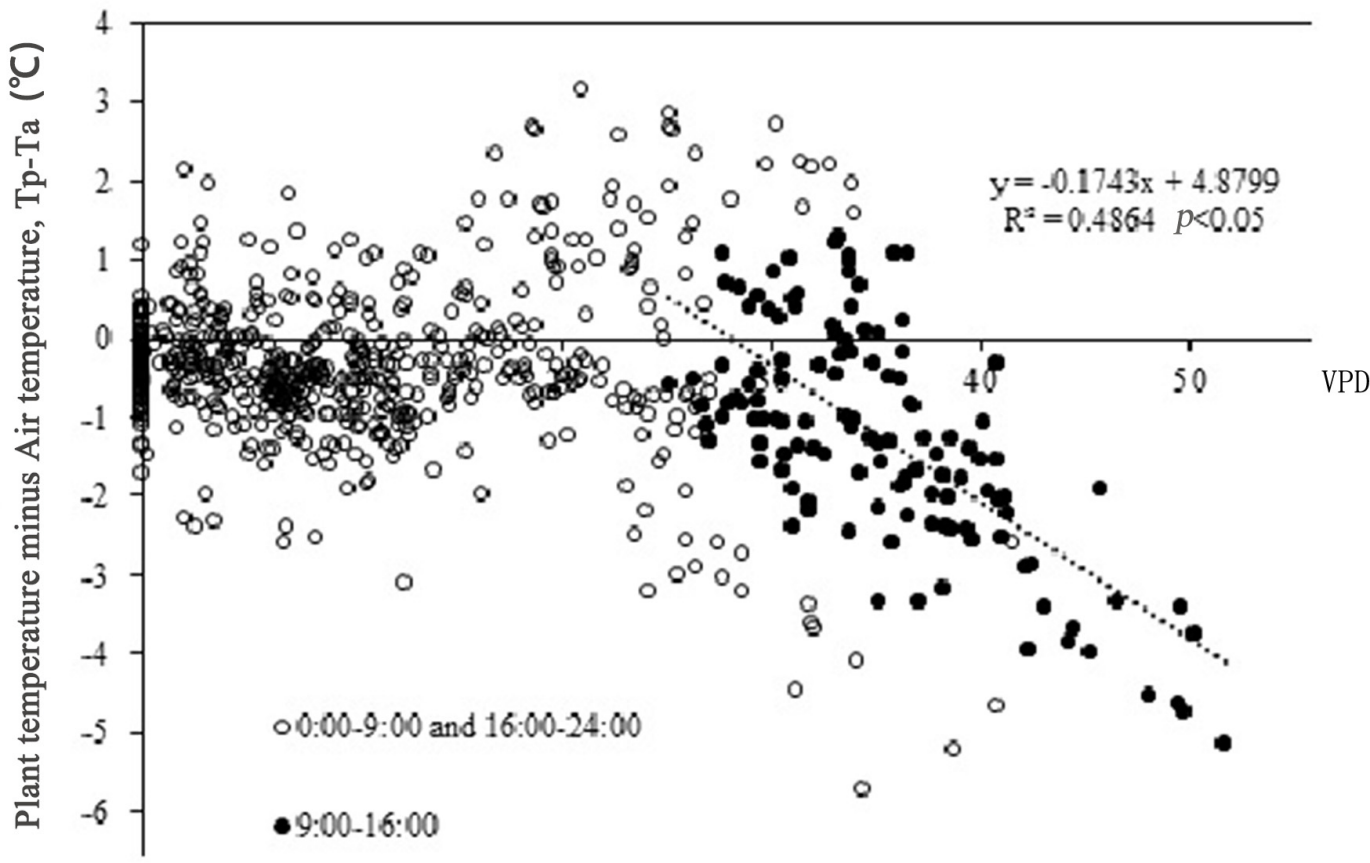
Correlations between vapor pressure deficit (VPD) and plant temperature (Tp) in *Artemisia ordosica*. A negative relationship between Tp and VPD was deleted based on data collected from 9:00–16:00 (closed symbols), however no significant difference was observed at other times of a day (open symbols).

Fig 5 shows the temperature differences between the stem and air. Tp is generally consistent with Ta at most times of the day, 0–2°C above Ta from 19:00 to 22:00, and almost identical to Ta at night. When Ta increases at midday, Tp-Ta appears to begin to decrease and Ta increases to become higher than Tp. Regression analysis of VPD and Tp–Ta (Fig 6) showed that from 9:00 to 16:00, as VPD was increasing, Tp–Ta decreased. VPD and Tp-Ta showed a negative linear correlation (P<0.05). However, at other times, VPD and Tp–Ta showed no clear correlation. This confirms that plants can trigger a self-cooling ability to lose heat when their surroundings become hotter and drier relative to the plant.

#### Changes of transpiration and correlations with Tp–Ta

**Fig 7:**
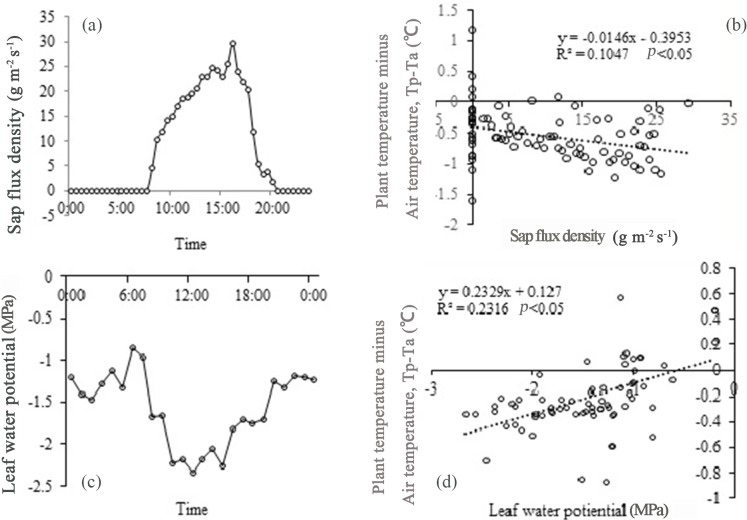
Diurnal variation in sap flow velocity & leaf water potential and relationships with plant-air temperature differences (Tp-Ta) in *Artemisia ordosica*. (a) Data from the day on 10 July 2014. (b) A negative relationship between sap flow velocity and Tp-Ta was detected (Sig. probability less than 0.05). (c) Data from the day on the same day with sap flow measurement. (d) A positive relationship between leaf water potential and Tp-Ta was observed (Sig. probability less than 0.05).

At midday, the high sap flow and low leaf potential result in intense transpiration (which forms a major portion of the latent heat) (Fig 7). Regression analysis between both Tp–Ta and sap flow velocity, and Tp–Ta and leaf water potential, indicated linear correlations with a high level of significance (P<0.05).

#### Changes in plant water conductance and correlations with Tp–Ta

**Fig 8:**
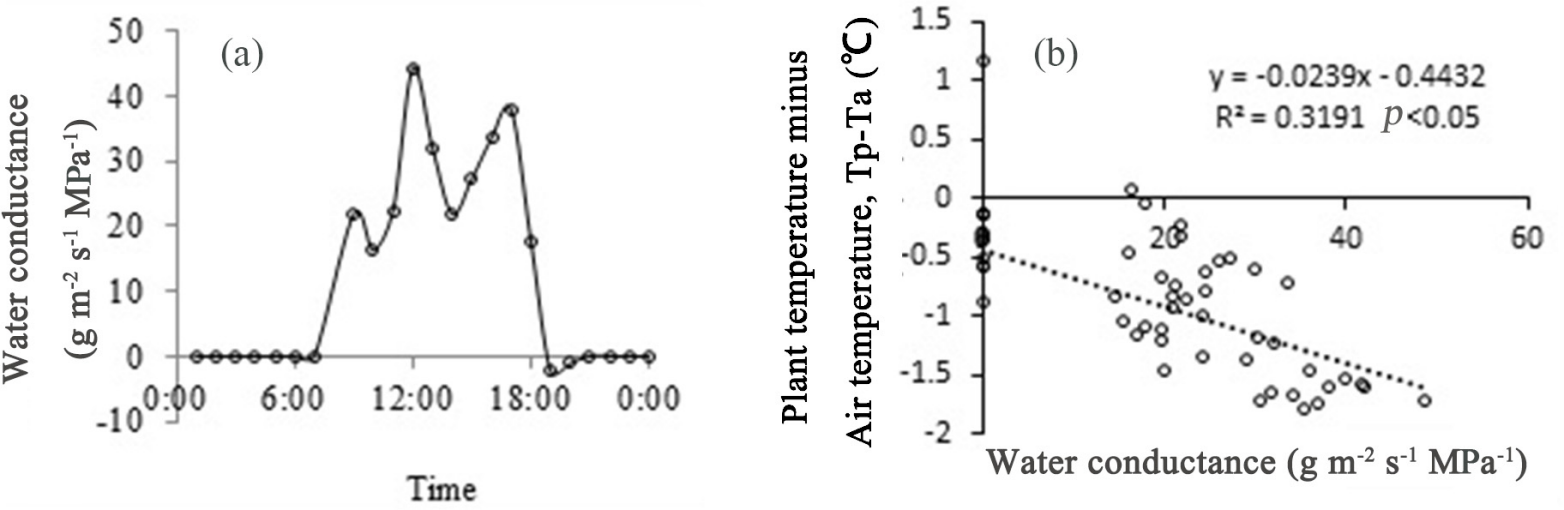
Diurnal variation in water conductance and the relationship with plant temperature (Tp) in *Artemisia ordosica*. (a) Water conductance was calculated using data collected on 10 July 2014. (b) A negative relationship between water conductance and Tp-Ta was detected (Sig. probability is less than .05).

Surprisingly, the stem water content abruptly increased at midday. We calculated the water conductance in the stem from the variation in sap flow and leaf potential; the results (Fig 8) showed that the conductance increased by about 100% at midday (around 12:00) compared to dawn (around 9:00). Regression analysis showed a well-defined negative correlation, indicating that the Tp–Ta decreased with increasing water conductance.

## Discussion

The data in this study concerned the self-cooling ability of plants, which has not been widely recognized to date. In addition, we measured variations in stem temperature to confirm this ability, which has not been attempted previously.

### Tp dynamics in the open field

Tp generally followed the spatial and temporal trends in Ta. Tp also varied with height because the solar radiation intensity received by the leaves and stems also differs by height. This was demonstrated by Brown and Covey, who inserted copper-constantan thermo-junctions into the undersides of leaves at five different heights on the plant [2]. In our study, the lowest height always showed the highest temperature in both the old and young branches at midday. The sand temperature suggested that the surface layer of the sand was heated by solar radiation and therefore had an important influence on the plants. During the day, solar radiation affected Tp from the top down, and radiation from the sand affected Tp from the bottom up. At night, the difference in Tp between different heights in the plant was small because there was no solar radiation and the sand temperature remained more constant.

Although the changes in Tp generally followed those of Ta, there were some considerable differences between the trends in Tp and Ta. It is widely accepted that the most sensitive part of the plant is its canopy [19, 20]. We also found that the leaves were heated after sunrise, but the stem temperature remained steady. This phenomenon is related to the thermal time constant, which provides a measure of how closely tissue temperature tracks Ta in a changing environment. The larger the thermal time constant, the more difficult it is to raise the temperature in the plant organs. Some studies have indicated that wind speed, Ta, size of the plant organs, and specific heat capacity are the main factors influencing the thermal time constant [21]. In our study, all plants were under a similar environment, so the effects of differences in the wind and Ta can be excluded. Because the thickness and specific heat capacity of the stem are larger than those of the leaves, which indicates a larger thermal time constant, the stem temperature is more stable. Thus, abnormal temperature changes in the stem would provide stronger evidence (compared with the leaves) of the plant’s response to its surroundings.

### Assumption of internal controls on plant self-cooling

While the empirical evidence provides little support for operational homeothermy, it has been shown that the Tp in the stem is fairly constant under normal, steady environmental conditions. By inference, the finite thermal conductivity of trunks or branches leads to slow transfer velocity from surface to interior and vice versa [22]. However, this was contradicted by the sudden slowing of the increase in stem temperature captured in our study, and we assumed that this phenomenon confirmed the self-cooling ability of plants.

One of the mechanisms of plant cooling ability is latent heat loss. It is interesting to note that when VPD increases at midday, latent heat dissipates; this is an important plant self-cooling mechanism (Fig 6). As the major contributor to latent heat dissipation, we evaluated transpiration in further detail. The sap flow and leaf potential data showed that transpiration was fairly intense at midday. Therefore, transpiration likely contributes to a plant’s ability to cool itself at high temperature. However, it has been reported that transpiration reduced leaf surface temperatures by only 20% of the shortwave absorption causing leaf surface temperatures to increase [23]. This suggests that plant heat release through transpiration is unable to counteract the effects of environmental heat stress. Thus, reduction in stem temperature must be mediated by other mechanisms.

Regression analysis showed that higher water conductance might be another mechanism of plant self-cooling ability. Regarding specific heat, the increase in water conductance of the stem leads to an increase in the water content of the stem. The higher stem water content increases the specific heat, which leads to a decrease in the thermal conductivity, preventing the temperature in the branches from increasing further [24,25].

In addition, the cold water drawn up into it from underground also cools the stem. We measured the soil temperature at depths of 10, 20, and 30 cm below the soil surface, which is the root zone. In these layers of sand, the soil temperature is considerably lower than that of the stem and air. Therefore, we hypothesize that sap flow transports water from the soil, decreasing the stem temperature, especially at midday when there is intense transpiration. This is another mechanism of plant self-cooling.

Although our data are incomplete, they support those of several previous studies. The self-cooling ability is a mechanism of plant adaptation to the environment, especially for avoiding heat damage. Effective functioning of the self-cooling mechanism reflects high adaption to the environment. Hence, we can apply knowledge of plant self-cooling to select species that are well adapted to certain environments for introduction and propagation. Moreover, such knowledge can in future be applied to plant temperature control for forest management.

Based on the results of studies with general circulation models of the atmosphere, it important to explore plant self-cooling ability in more detail and develop methods such as controlled tests [26]. Further field studies in other plant species and under a variety of environmental conditions are required [27].

## Conclusions

Variations in Tp are highly dependent on Ta. The self-cooling ability of plants was confirmed, especially at midday. The self-cooling phenomenon reflects a mechanism of the plant self-regulating system, and is regulated by the transpiration rate, increase in stem water conductance, and the uptake of cool soil water.

This study will facilitate vegetation management in terms of avoiding thermal stress in plants. Moreover, our findings will assist species selection with taking plant temperature as an index.

## Supporting Information

S1 Dataset The Excel sheet “S1” shows all data used in this paper, including plant temperature, microclimate factors and physiological characteristics. Worksheets are arranged in order of appearance of the corresponding Figures. Each worksheet has been clearly titled and the labels in each rank are marked.
(XLSX)
